# Sympathetic Ophthalmia – case report

**DOI:** 10.22336/rjo.2022.17

**Published:** 2022

**Authors:** Sorin Simion Macarie, Diana Huțu

**Affiliations:** * Department of Ophthalmology, University of Medicine and Pharmacy, Cluj-Napoca, Romania; ** Department of Ophthalmology, Cluj County Emergency Hospital, Cluj-Napoca, Romania

**Keywords:** sympathetic ophthalmia, ocular trauma, vision loss, enucleation

## Abstract

**Purpose.** To present the case of a 22-year-old man with a history of trauma on the right eye, followed by a sudden decrease of visual acuity on the left eye, but with a good recovery after surgical treatment.

**Material and methods.** We reported a case of a 22-year-old patient with a sudden and painless decrease of visual acuity on the left eye, a month after a car accident, which led to the laceration of the right globe. At first, the patient received only medical treatment because he refused any surgical intervention. He had a favorable evolution during hospitalization, but he returned after a month with the same visual acuity as at his first admission. The patient accepted the medical treatment and the enucleation of the right eye, thus having a fast improvement in his visual acuity on the left eye.

**Conclusions.** Although the enucleation was overdue, it had a strong favorable influence on the evolution of the disease. As a result of the surgery, the visual acuity has improved significantly in just a few days.

**Abbreviations:** OCT = optical coherence tomography

## Introduction

Sympathetic ophthalmia is a rare, bilateral diffuse granulomatous intraocular panuveitis that occurs in most cases within days or months after penetrating trauma, in some cases with uveal prolapse or less frequently after intraocular surgery, such as vitreoretinal procedures. The injured eye is red and irritable and the sympathizing eye develops loss of visual acuity, photophobia, and loss of accommodation [**1**,**2**].

The anterior uveitis develops in both eyes, but the severity of inflammation may be significantly asymmetrical and it is represented by keratic precipitates, anterior chamber flare and posterior synechiae.

The fundus of the sympathizing eye may present papillitis, multifocal choroidal infiltrates in the midperiphery, exudative retinal detachment and vasculitis [**3**,**2**].

The pathological mechanism of the sympathetic ophthalmia is an autoimmune reaction directed against cells that contain melanin in the uvea. After the ocular trauma, the blood-retinal barrier is disrupted, exposing ocular antigens (melanin) to the conjunctival lymphatics, and being leaked into the systemic environment. The damaged tissue alerts and recruits antigen presenting cells to the site of trauma where they phagocytose and process the ocular antigens that are no longer recognized as “self” components. This T-cell-mediated immune response creates anti-uveal antibodies and triggers an inflammatory reaction in both eyes [**4**-**6**].

Histopathology in sympathetic ophthalmia shows a diffuse lymphocytic infiltration of the choroid and aggregates of epithelioid cells that contain fine granules of melanin.

 There may be systemic manifestations that are similar to those in Vogt-Koyanagi-Harada Syndrome, but they are very rare. The prognosis depends on several factors, such as location of disease, the severity of the lesions and the response to treatment. Complications that may occur include cataract, glaucoma, optical atrophy and chorioretinal scarring [**7**,**2**].

The treatment is represented primarily by high dose oral corticosteroids initially, followed by a gradually tapered dose according to patient’s evolution. The enucleation of the injured eye in the first week following the injury is also an option that showed significant favorable effects in some cases, but it remains a controversial approach [**2**,**6**].

## Case report

We present the case of a 22-year-old patient, with a sudden and painless decrease of visual acuity on the left eye, without any other symptoms, a month after the traumatic laceration of the right globe. He denied the use of toxic substances and he had no allergies. After the initial right eye trauma, the surgical intervention consisted of the suture of the laceration of the right eye, the excision of the herniated uvea and the suture of the conjunctiva, but also the lid margin.

The assessment of vision revealed no light perception in the right eye and counting fingers at 30 cm in the left eye. The intraocular pressure of the right eye assessed by digital palpation was low and in the left eye was 10 mmHg. 

**Fig. 1:**
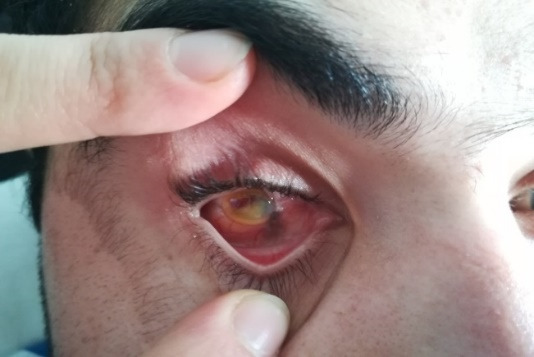
Right eye aspect

The examination of the anterior segment of the right eye showed an atrophic globe, a cornea with a diameter of approximately 8.5 mm with complete opacification and blood infiltrate, limbus sutures at 12 and 6 o’clock infiltrate (**Fig. 1**). The left eye revealed a conjunctival congestion, a few small keratic precipitates, positive Tyndall reaction, miosis and a posterior synechiae at 10 o’clock. 

The fundus in the right eye could not be examined and, in the left eye, it presented difficulty papillary edema and a yellow retina, completely detached in all quadrants.

**Fig. 2:**
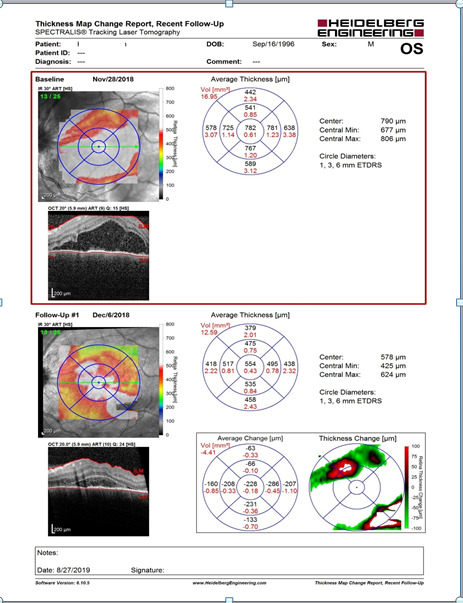
OCT examination of the left eye macula

Three days after the admission, an OCT examination of the macula was performed, which confirmed the exudative retinal detachment (**Fig. 2**).

**Fig. 3:**
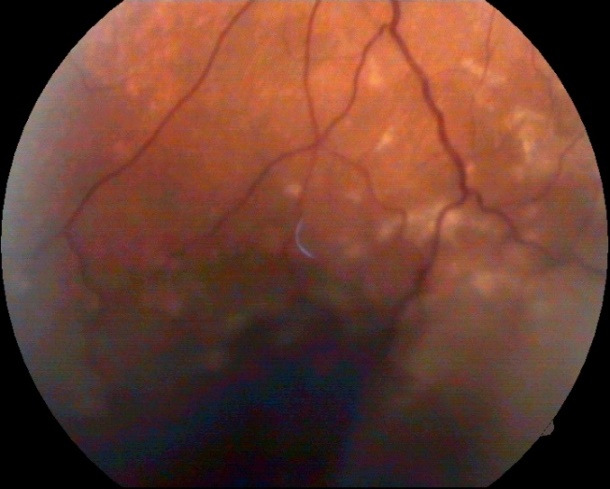
Fundus in the left eye one week after admission

A week after the admission, the fundus in the left eye revealed multifocal choroidal infiltrates (**Fig. 3**).

The general clinical examination showed no other pathological findings. At this point, the stage diagnosis was: left eye panuveitis with the suspicion of sympathetic ophthalmia, and exudative retinal detachment, and right eye post-traumatic atrophic globe.

The hematology tests revealed a mild increase of white blood cells, with no other findings and the serological tests for syphilis were negative. The IDR tuberculin test was negative and the negative or non-reactive immunology findings were: anti-Borrelia IgM and IgG Ab, p-ANCA and c-ANCA Ab, anti HBs Ab, HBs Ag, anti HCV Ab, HIV Combined test.

The skull CT scan of the right eye showed a globe a hyperdense appearance, mostly in the vitreous body, with extension to the retinochoroidal layer, a small dimension of the globe and infiltrated retrobulbar fat. The optic nerve and extrinsic muscles were in normal parameters, as well as the skull and chest radiography. 

The general treatment consisted of high dose Methylprednisolone, tapering the dose by half each week, Ceftriaxone for ten days and Metamizole when needed. The local treatment was represented by tropicamide eye drops and a fixed combination of betamethasone and chloramphenicol eye drops. The patient was advised to consider the enucleation of the right eye, but he refused.

**Fig. 4 A, B:**
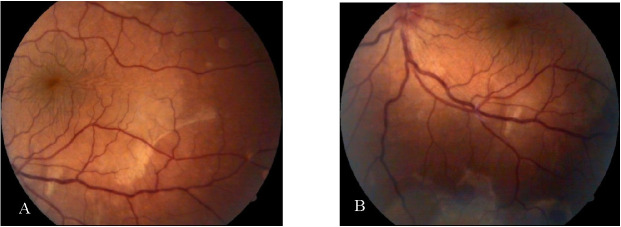
Fundus in the left eye at the end of hospitalization

The evolution of left eye during hospitalization was favorable: visual acuity improved every week from 1/ 50, 3/ 50 to 0.3 on the day of discharge. The anterior segment showed a clear cornea and anterior chamber, with a few central pigment depositions on the anterior lens capsule. The left eye fundus showed a gradual improvement, with progressive retinal attachment from the superior to the inferior quadrants (**Fig. 4 A, B**). At discharge, no evidence of retinal detachment was present except for a mild papillary edema.

The patient continued the oral treatment with Methylprednisolone at home, tapering the dose with one tablet per week and local treatment with nonsteroidal anti-inflammatory eye drops. 

**Fig. 5:**
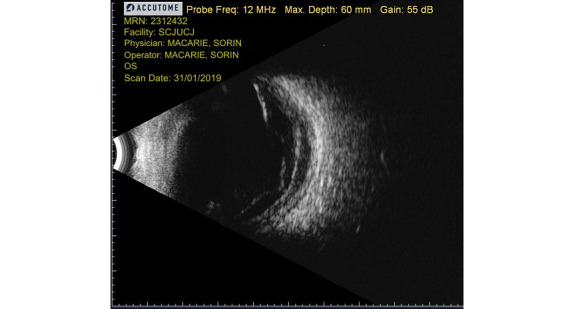
B-scan ultrasound, left eye

After another month, the patient returned with a sudden and painless decrease of left eye visual acuity. The anterior segment examination revealed similar findings to the first admission, except this time the posterior synechiae were on almost 360°. The fundus on the right eye could not be examined and the retina was again detached on the left eye, which was confirmed by B-scan ultrasound (**Fig. 5**). We appreciated that the positive diagnosis was sympathetic ophthalmia in the left eye.

**Fig. 6:**
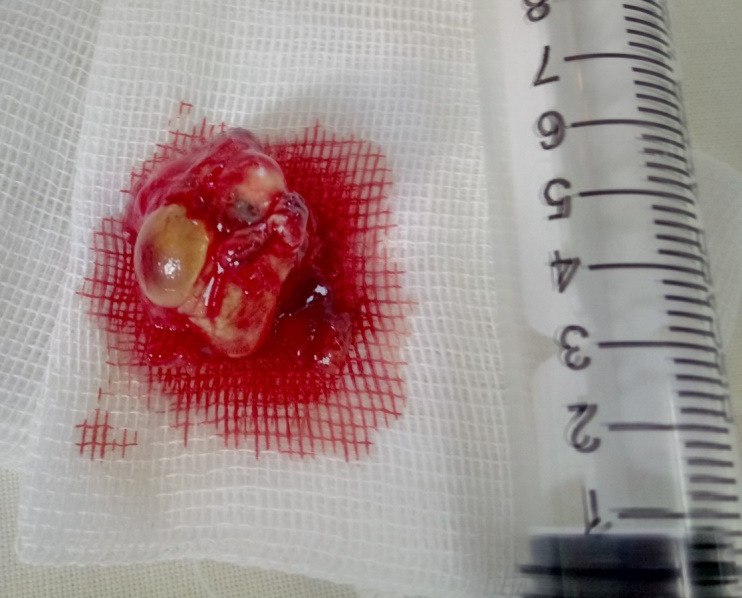
Aspect of the enucleated right eye

The patient followed the same medical treatment and furthermore, he accepted the enucleation of the right eye. **Fig. 6** presents the aspect of the enucleated right eye. The histopathology report confirmed our diagnosis.

**Fig. 7 A,B:**
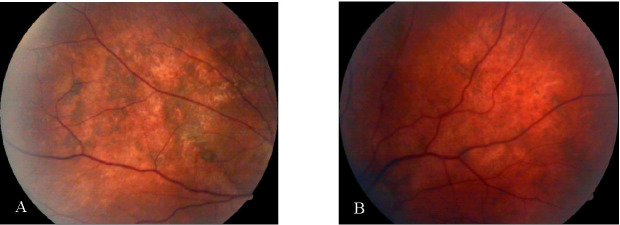
Left eye fundus aspect after enucleation of right eye

After the enucleation, the left eye visual acuity improved to 0.2, the only findings at the anterior segment being represented by several depositions on the anterior lens capsule and at the fundus, the retina being only inferiorly detached with multiple pigment migrations (**Fig. 7 A, B**).

**Fig. 8:**
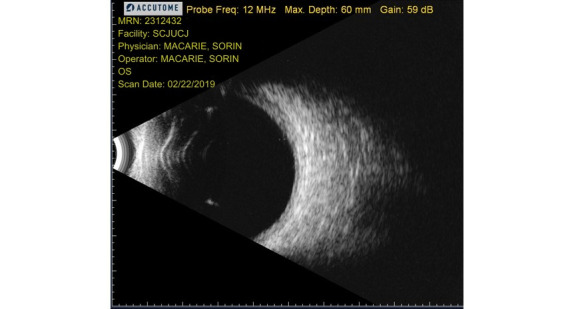
Final B-scan aspect of the left eye

After a week, retinal laser photocoagulation was performed in the left eye, which improved the visual acuity to 0.3 and the left eye retina was completely attached (**Fig. 8**).

## Discussions

Without treatment, sympathetic ophthalmia can lead to blindness with optic atrophy and persistent retinal detachment. The medium- and long-term prognosis is favorable under high dose immunosuppressant medication, which is gradually tapered. The visual acuity at discharge can be maintained, but the risk of the inflammatory process cannot be excluded.

 The inflammatory signs were more advanced at the posterior segment than the anterior segment. During the first hospitalization, the evolution was favorable at first under medical treatment, but with a quick recurrence due to the patient’s refuse of the surgery. Although the enucleation was overdue and it is considered a controversial approach, it had a strong favorable influence on the evolution leading to a significantly improved visual acuity in just a few days.

## Conclusions

Sympathetic ophthalmia remains a problem in ophthalmology practice, especially after major open ocular trauma with large ocular lacerations. In those cases, initial trauma produces severe eye injuries, with major ocular content loss. Evolution is often toward eyeball atrophy. All these factors can lead to development of sympathetic ophthalmia. Some patients do not agree with atrophic eye enucleation, so the occurrence of sympathetic ophthalmia can produce important vision disturbance in the fellow eye.

**Conflict of Interest statement**

Authors state no conflict of interest.

**Informed Consent and Human and Animal Rights statement**

Informed consent has been obtained from all individuals included in this study.

**Authorization for the use of human subjects**

Ethical approval: The research related to human use complies with all the relevant national regulations, institutional policies, is in accordance with the tenets of the Helsinki Declaration, and has been approved by the review board of Cluj County Emergency Hospital, Cluj-Napoca, Cluj.

**Acknowledgements**

None.

**Sources of Funding**

None.

**Disclosures**

None.
